# Seizing the strategic opportunities of emerging technologies by building up innovation system: monoclonal antibody development in China

**DOI:** 10.1186/s12961-015-0056-1

**Published:** 2015-11-04

**Authors:** Mao-Yu Zhang, Jian Li, Hao Hu, Yi-Tao Wang

**Affiliations:** State Key Laboratory of Quality Research in Chinese Medicine, Institute of Chinese Medical Sciences, University of Macau, Room 2057, Building N22, Avenida da Universidade, Taipa, Macao, China; Faculty of Arts and Humanity, University of Macau, Macao, China

**Keywords:** China, Emerging technology, Innovation system, Monoclonal antibody

## Abstract

**Background:**

Monoclonal antibodies (mAbs), as an emerging technology, have become increasingly important in the development of human therapeutic agents. How developing countries such as China could seize this emerging technological opportunity remains a poorly studied issue in prior literature. Thus, this paper aims to investigate the research and development of mAbs in China based on an innovation system functions approach and probes into the question of how China has been taking advantage of emerging technologies to overcome its challenges of building up a complete innovation system in developing mAbs.

**Methods:**

Mixed research methods were applied by combining archival data and field interviews. Archival data from the China Food and Drug Administration, Web of Science, the United States Patent and Trademark Office, the Chinese Clinical Trial Registry, and the National Science and Technology Report Service were used to examine the status quo of the technology and research and development (R&D) activities in China, while the opinions of researchers and managers in this field were synthesized from the interviews.

**Results:**

From the perspective of innovation system functions, technological development of mAb in China is being driven by incentives such as the subsidies from the State and corporate R&D funding. Knowledge diffusion has been well served over the last 10 years through exchanging information on networks and technology transfer with developed countries. The State has provided clear guidance on search of emerging mAb technologies. Legitimacy of mAb in China has gained momentum owing to the implementation of government policies stipulated in the “The Eleventh Five-year Plan” in 2007, as well as national projects such as the “973 Program” and “863 Program”, among others. The potential of market formation stays high because of the rising local demand and government support. Entrepreneurial activities for mAb continue to prosper. In addition, the situation of resource supply has been improved with the support of the State.

**Conclusions:**

This study finds that a complete innovation system for mAb has begun to take shape in China. MAb innovators in China are capitalizing on this emerging technological opportunity to participate in the global drive of developing the value chain for the innovative drug. In the long run, the build-up of the research system for mAb in China could bring about more driving forces to the mAb innovation system.

## Background

Over the past three decades, monoclonal antibodies (mAbs) have made a dramatic transformation from scientific tools to powerful human therapeutic agents [1]. Sales of mAb therapies exceeded 40 billion US dollars in 2010 and are expected to reach 70 billion US dollars by 2015 [2]. In 1975, Koehler and Milstein first described the in vitro production of murine mAbs from hybridomas [3]. In the late 1980s, clinical development of murine mAbs was initiated but then inhibited by numerous significant drawbacks [4]. Later, in an attempt to overcome the inherent immunogenicity concerns and the reduced effect or function of murine mAbs in human [5], chimeric mouse-human antibodies were developed [6]. Nowadays, humanized mAbs are the fastest growing category of mAb therapeutics entering clinical study [7]. Development of this class of therapeutic agents started as early as 1980s but achieved no clinical or commercial success until 2002, when adalimumab became the first humanized mAb approved by the United States Food and Drug Administration [8]. Thus far, a total of seven humanized mAbs have been approved for marketing in the United States.

While mAbs, as an emerging technology, have become increasingly important in the development of human therapeutic agents, how developing countries such as China could seize this emerging technological opportunity remains a scantly studied topic in prior literature. Aiming at investigating mAb development in China by drawing on an innovation systems approach, this paper probes into the question of how China has been taking advantage of emerging technologies to overcome challenges through building up an innovation system in developing mAbs. In so doing, it attempts to contribute to (1) an understanding of the establishment and structure of the mAb innovation system in China; (2) an evaluation of the performance of the mAb innovation system by analysing the functions of the innovation system; and (3) providing policy suggestions for improving the mAb innovation system.

Further, this paper offers an overview of the functions of innovation system approach, which provided the theoretical framework for examining the mAb innovation system, and analyses the innovation system of mAbs and the function fulfilment. To deepen the theoretical understanding, we discuss the research findings by comparing the Chinese mAb innovation system with that of the United States and India, as well as with innovation systems of Chinese pharmaceuticals, concluding with lessons from the managerial and policymaking perspective.

## Theoretical framework

Emerging technologies, such as mAbs, are expected to contribute to technology advances, economic development, and environmental and health improvements [9–14]. However, compared with traditional technologies, emerging technologies are newly introduced, fast changing and developing, and have relatively limited applications in the market, which is characterized by greater measures of uncertainty [15–18]. Therefore, the development and commercialization of emerging technologies are heavily dependent on the establishment and operation of an innovation system [19].

For an emerging technology, the idea of a technological innovation system was considered as a new method to understand the dynamic changes of technological progress [20]. The technological innovation system is regarded as (1) “*a heuristic attempt, developed to analyse all societal subsystems, actors, and institutions contributing in one way or the other, directly or indirectly, intentionally or not, to the emergence or production of innovation*” [21, 22]; and (2) a systematic combination of actors, networks, and institutions [23, 24]. Therefore, the framework of technological innovation systems provides an effective means to study emerging technologies such as mAb.

Traditional innovation systems studies mainly concentrate on the system structure, which has been proven to be insufficient to identify policymaking recommendations [25]. Thus, an in-depth analysis of innovation systems is warranted to understand the influence of a series of processes called “functions” [25]. Once known what innovation systems functions promote or hinder the innovation, policies and management activities that are necessary for improving the innovation system can be easily identified [26]. Innovation systems functions are interpreted in seven dimensions, as indicated below [27–29]:

- *Function 1: Entrepreneurial activities*
  Entrepreneurs are of prime importance; without entrepreneurs, no innovation system would take place. What the entrepreneurs do is turn the potential of a new technology into concrete action and take full advantage of business opportunities [30].
- *Function 2: Knowledge development (learning)*
  If solutions to identified problems are to be provided, new technologies must be developed. Research and development (R&D), search and experimentation, learning-by-doing/using and imitation are regarded as possible sources of new technologies. They may combine old and new technologies in innovative ways and reuse old knowledge by imitation [31].
- *Function 3: Knowledge diffusion through networks*
  Exchanging information is the essential characteristic of networks, such as changing norms and values. The diffusion may lead to a change in R&D agendas [32].
- *Function 4: Guidance of the search*
  Activities can positively affect the visibility and clarity of specific needs [30]. Expectations are also included.
- *Function 5: Market formation*
  It is difficult for emerging technologies to compete with embedded ones. Therefore, it is important to create protected markets for new technologies by formation of temporary niche markets and tax regimes of minimal consumption quotas [33].
- *Function 6: Resource mobilization*
  Both finance and human capital are necessary for all the activities within innovation systems [21]. They may determine the success or failure of a project.
- *Function 7: Advocacy coalition (creation of legitimacy/counteract resistance to change)*
  In order to become a well-developed technology, emerging technologies must be part of an incumbent regime. Parties with vested interests will often oppose the force of ‘creative destruction’. Thus, advocacy coalitions can function as a catalyst to place a new technology on the agenda. If successful, advocacy coalitions grow in terms of size and influence and can become powerful enough to brisk up the spirit of creative destruction [22].

## Methods

In an attempt to provide a comprehensive research of mAbs development in China, this study employed a multi-dimensional design to enhance the rigor and relevance of the study [34–36]. Thus, multiple data sources are used to serve the purpose. Basically, our approach consists of retrieving a wide range of activities using a variety of sources related to the development and diffusion of the technology under study.

Firstly, archival data were collected from multiple sources, as listed below.

- *Product registry*
  Database in China Food and Drug Administration was searched to confirm the exact number of domestic mAb products approved in China. “Biopharmaceutical drugs” as a category was selected in “domestic products”, and “monoclonal antibody” was used as a keyword; diagnostic antibodies and diagnostic agents were excluded.
- *Patent*
  The search strategy was defined as the mAb patents applied by Chinese Applicants in recent decades in the United States Patent and Trademark Office. Patent information with satisfactory purposes was searched using the following strategies: Topic = (monoclonal antibod*) AND Title = (monoclonal antibod*) AND Assignee = (CHINA). There was no limit to the year.
- *Publication*
  Academic publications related to mAbs in China were extracted from the Thomson Reuters’ Web of Science database. Studies and research about mAbs in China began in the 21st century [37]. Publications related to mAbs between 2000 and 2013 were searched using the following strategies: Topic = (monoclonal antibod*) AND Title = (monoclonal antibod*) AND Address = (China not Hong Kong not Taiwan not Macau). “mAbs” was used as the keyword in the same way. In the query above, the asterisk (*) represents any group of characters or no character and the literature type was limited in “Article”. Given a better understanding about the position of China’s mAbs in the world, publication status of anti-tumour mAbs in China was chosen as an example and compared with “Top 5” countries with respect to mAbs. While keeping the database updated, all the data used were as recent as until March 21, 2014. Selected documents included “monoclonal antibod* or mab*” in the title or “monoclonal antibod* or mAb*” in the topic, and “cancer or tumor or anti-cancer or anti-tumor” in the topic. “Address” searched for the selected countries and regions, including China (excluding Hong Kong, Taiwan and Macau), United States, Japan, Germany, United Kingdom, Italy, France, the Netherlands, and Canada. The document type was limited in “Article” as well. The publication years covered were from 1980 to 2013, since commercially sponsored mAbs began entering clinical study in 1980 [38].
- *Clinical trials registry*
  The Chinese Clinical Trial Registry database was the source used to present situations about assignees and funding of clinical trials applied in China on mAbs. Both Chinese and English databases were used to search and sort records. “mAb” and “monoclonal antibody*” were used as keywords; the year was not limited.
- *Research projects*
  The National Science and Technology Report Service database, which offers open access, was searched to ascertain and narrow down the list of targeted key mAb institutions.

In addition, field interviews were conducted to collect the relevant information. Expert, rather than general or informal, opinions play a crucial role and are often sought for professional advice in the development of technology and policy change to enhance knowledge of emerging technologies such as the mAb industry, even though the information may reveal the risks, benefits and regulations involved [39, 40]. For the purpose of this study, a series of interviews were conducted with six Chinese researchers specialized in mAbs and managers from three leading Chinese mAb firms. The interviewees were identified through the National Science and Technology Report Service database. The interview design was reviewed and approved by the Ethics Committee of the University of Macau. The chosen interviewees were contacted through email first to obtain their consent. The interview questions did not involve confidential information but were just designed from the perspectives of technology, the characteristics and strategies of R&D, and the challenges and technical obstacles, among others. In addition, some documentary materials were also referred to [41–43].

With all the material collected from the multiple sources described above, we conducted the data analysis in two stages. First, following the components of technological innovation system, we categorized the materials into four aspects: mAb technology development, mAb firms, research institutes and their networking with firms, and institutions. All the quantitative and qualitative materials were used as complementary and used to cross-check our findings. Second, based on the results of the first stage, we analyzed the functions of mAb innovation systems in terms of the seven dimensions as defined above. Such an analysis framework for technological innovation system has been widely validated in the study of emerging technologies, and it is therefore appropriate to apply this kind of analysis framework to analyse mAb innovation systems. The final results of analysis are reported in the next two sections.

## mAb innovation systems in China

### Stages of mAb technology development in China

mAbs in China were originally developed from the tracking of existing technologies. Having prepared itself for the new opportunity on a theoretical basis, China commenced engagement with researchers and developers of other countries in order to allow the cutting-edge technology to flow in directly. Into the 21st century, Chinese mAb R&D finds itself at a more mature stage with an established production platform and patented innovative drug activities impending. The development of mAbs in China could be summarized as consisting of the following stages:

- *Absorbing stage*
  Tracking the cutting-edge technology from foreign countries was the objective at this stage, with the advent of its own publications on the existing foreign mAb agents as well as the local R&D processes. China was preparing the first domestic mAb products for introduction into the market.
- *Exploratory stage*
  By buying and the introducing the technology, domestic products were listed on the market. By the year 2006, four local agents had been developed, including “me-too” and “me-better”. The number of publications concerned was accumulating. Literature types not only included reviews but also new-phased research results. At this stage, China started performing joint research with developed countries such as the United States to explore the path to independent studies.
- *Innovation stage*
  At this stage of China’s mAb evolution, a total of eight domestic products were available, some of which had reached the stages of humanized technology. The mAb agents were gradually expanding with abundant indications in the process. The R&D institutes attempted to push for United States Patent and Trademark Office approval of their domestic products and succeeded in 2006. The content of the patents in general was concentrated on the diagnosis of major diseases such as cancer and also on mAb preparation and production methods. The number of clinical trials for second and third generation anti-cancer products increased rapidly. Moreover, it was found that enterprises whose products had been put into the market had invested more effort and funding in clinical trials, hence becoming the main sponsors in China.

### mAb firms in China

While there are still few original innovation drugs developed in China, the status quo of foreign players dominating the Chinese mAb market has now changed. R&D of mAbs in China began in the 1980s, but it was not until 1999 that the first mAb therapeutic agent was introduced. To date, there are seven domestic companies with eight products launched. Of the seven, four are producing “me-too” or “me-better” drugs (Table 1). However, the products have no property rights of their own. These small and medium-sized enterprises are typically with of a fragile foundation and early-stage high R&D costs, as a result of which they cannot afford the cost of long-term clinical trials, but rely heavily on imported technology. Thus, most focus on manufacturing instead of technology development aiming at the domestic market only.

**Table 1:** Leading actors (industry) of mAbs in China

Company	Year	Ownership	Registered capital (Million)	Primary products	Indications	Medical insurance	R&D	Market	Position	Strategy
Bio-tech	2000	Private	115.38	Taixinsheng	Nasopharyngeal cancer	No	Me-better	Export, domestic market	Hospitals	Academic promotion
Shanghai CP Guojian pharmaceuticals	2002	Private	686	Jiannipai	Kidney transplants	No	Me-better	Export, domestic market	Comprehensive and specialized hospitals	Academic promotion
				Yisaipu	Rheumatoid arthritis, psoriasis and Ankylosing spondylitis	Yes				
Huasun	2005	Private	127.98	Licartin	Liver cancer	No	Me-too	Domestic market	Therapeutic centre	Academic promotion

The rest, led by Shanghai CP Guojian pharmaceuticals (CPGJ), have been growing steadily and trying to transform “following” into “innovation”. Therefore, they take full advantage of pertinent policies and national research projects to organize future-proof R&D activities. Their development path represents the future direction of Chinese mAbs.

- Taixinsheng, produced by Bio-tech pharmaceuticals and approved in 2008 had been the first outcome of national-level projects among developing countries. Substantial support was provided by the government program between China and Cuba, thus Bio-tech performed as an important cooperative carrier of R&D technology on nasopharyngeal cancer.
- Based in east Shanghai, Shanghai CPGJ, was founded in 2002 and was jointly invested by China International Trust and Investment Corporation and Shanghai Lansheng Guojian pharmaceutical company limited. CPGJ made a joint R&D effort with the Second Military Medical University, one of earliest Chinese military universities. CPGJ’s products include mAb (Jiannipai, developed for kidney transplantation) and fusion protein (Yisaipu, developed for rheumatoid arthritis, etc.). Yisaipu has been successfully listed in the National Medical Insurance of numerous provinces and has consequently earned large-scale government orders. Further, CPGJ owns the intellectual property rights solely.
- Huasun Biotech, established in 2005, is a subsidiary of the Chengdu Huasun Biological Technology Company Limited with listing in Shenzhen since 1998. Huasun Steel Structure, another Huasun subsidiary, provided a steady and stable inflow of cash. Moreover, Huasun Biotech seized the chance to enter the biopharmaceutical market by technology transfer from the Fourth Military Medical University. The product, Licartin, is the first domestic mAb for hepatocellular carcinoma.

While the domestic actors imported and absorbed a mature technology, the price of domestic mAb products still remains high, which suggests enormous mass production costs for the local manufacturer. Therefore, improving the capacity for efficient and advanced antibody expression is conducive to cutting the costs.

Apart from CPGJ and Bio-tech, the remaining companies mainly concentrate on the domestic market at present. On the one hand, the demand of antibody-based drugs is rather high, whereas producers of this kind of therapy agents are far from being adequate. On the other hand, their overall performance and competitiveness are just as inadequate for exportation of their products. To change this and open up international markets in the future, it is imperative for both policymakers and corporations that actions should be taken at this juncture.

### Research institutes and their networking with firms

In 1980s, China began implementing mAb research activities between the leading academic institutions at the national level. Led by the Chinese Academy of Sciences, the R&D institutions that were first established in 1949 when the Republic was founded, mostly with a military background, are centred on the upstream and middle reaches of leading antibody technologies. Their research areas are gene cloning, cell engineering and platform establishment, etc. For instance, CPGJ has ongoing partnerships in key technologies with the Second Military Medical University for mAb joint development. Successes in marketing Licartin are owed to the foundational technology transfer from the Fourth Military Medical University to Huasun in 2000.

The establishment of the institutions dates back to the last century. There has been great progress made in recent years, with bountiful results achieved by the institutions. They focus on R&D of upstream technology and transfer the basic technology to enterprises for production (Table 2), thus achievements from academic institutions make up the majority of the new drugs (Licartin and Yisaipu are a case in point). Meanwhile, academies are facing difficulties such as those associated with humanized antibodies and purification in the development of mAbs. To overcome the obstacles and lead to a brighter future, support from the government is deemed essential.

**Table 2:** Latest projects report related mAbs in 2013

Project	Institution of first author	Keywords
Humanized and human mAbs structure and antibody optimization techniques	Academy of Military Medical Science	Antibody humanized, human antibody, expression system, analysis system
Final report of tumour and autoimmune disease of certain target antibody drug design	Academy of Military Medical Science	Molecular simulation, molecular docking, target, BLyS, DR5, TNF
Antibody engineering drugs and synergistic technology	Chinese Academy of Medical Sciences	Antibody engineering drugs, immune coupling objects, antibody fusion protein, synergistic technology
Tumour marker optimization and clinical research and protein chip development	Second Military Medical University	Tumour marker, detection, protein chip, breast cancer, pancreatic cancer, primary liver cancer, colorectal cancer
Targeted complement inhibitor for systemic lupus erythematosus (SLE)	The People’s Liberation Army Institute for Disease Control and Prevention	SLE, alexin, CR2, targeted inhibition, physiology of immune defence
Studies on the novel technologies and approaches for tumour immunotherapy	Fourth Military Medical University	Tumour, immunotherapy, tumour vaccine, erbB2/HER2, apoptosis, exosome
Novel antibodies in the therapy of autoimmune disease	Second Military Medical University	Auto-immune disease, antibody drug, clinical therapy, mechanism investigation
Study on the new methods for diagnosing nasopharyngeal carcinoma in early stage	Sun Yat-Sen University	Nasopharyngeal carcinoma, Epstein-Barr virus, tumour biomarker, Bmi-1, CNEPF, LMP2A, IFI27
Immunological recognition, immune regulation and basic research of related immune diseases	Second Military Medical University	Immunological recognition, immune tolerance, related immune diseases
Common malignant tumour prevention, early detection and comprehensive treatment research	Sun Yat-Sen University	nasopharynx cancer, screening, early detection, Epstein-Barr virus, pathogenesis
Research and development of novel tri-specific single chain antibody drug for the treatment of ovarian cancer	Tianjin International Biomedical Research Joint Research Institute	Ovarian, tumour, antibody drug, specific
Passing report of 973 project “personalized immunosuppression plan of transplant patients”	Huazhong University of Science and Technology	Galectin-7, galectin-9, SNP, MDR1, IL-6, rejection, immune tolerance, proteomics
Acceptance report of basic research on organ transplantation immunology & application	Zhejiang University	Organ transplantation, transplantation immunology, chronic dysfunction, transplant infections, immunosuppression

Information retrieved from the National Science and Technology Report Service (www.nstrs.cn) database (until December 2013).

On the one hand, the government encourages domestic academies to take full advantage of national research projects and to cooperate with domestic enterprises. On the other, it explores new opportunities to connect with the leading foreign players for resource sharing. For instance, Institute Pasteur of the Shanghai Chinese Academy of Science was co-built for the purpose of biotherapeutics by the Chinese Academy of Sciences, Shanghai Municipality, and Institute Pasteur (France) in 2004.

The research-oriented institutions are making efforts to catch up with the advanced technology and work with businesses for industrialization. However, at present, few domestic products have had successful sales in China. The interaction between academies and enterprises is a rarity; as a result, most scientific research findings could not be translated into productivity. Although the organic integration of enterprises, universities and research institutes is recognized as a suitable mechanism and an economic developing mode for the biopharmaceutical industry in China, it is contingent on the support by the policy for the mAbs industry to open up its avenue for rapid development.

### Institutions

Policies concerning mAbs are always under the guidance of the government with respect to R&D in biotechnology. Since 1987, Chinese authorities in the field of biotechnology have issued a series of policies to promote and standardize the development of the whole industry. The main government agency in charge of policymaking is the State Council and its subsidiary National Development and Reform Commission, along with the Ministry of Science and Technology. Early key drivers, such as The Seventh Five-Year Plan, aimed at laying a foundation for further technology development; whereas the “863” Program (in 1986) and the “973” Program (in 1997) aimed at tracking industry development trends of developed countries at the strategic level. During the past 20 years, China has set clear targets for and a political commitment to the biopharmaceutical industry with major progress made in the current century.

Over nearly two decades, several state policies have been issued to support business finance, drug approval, marketing, and R&D in the biopharmaceutical industry. The policy direction of basic subjects like genome engineering and proteomics paved the way for mAbs development. Under the guidance of the “863” (in 1986) and the “973” (in 1997), and since the beginning of The Eleventh Five-year Plan, the antibody industry has been regarded as a major national innovative project, with its strategic position being thus formally established. It was the same with the developing path of the biological industry in that mAb development was spearheaded by academic institutions to be gradually transformed into enterprises in the ensuing years.

In 2006, the State called upon enterprises to take part in the development of the technology by putting forward a series of ordinances, programmes and policy changes, including the “National Program for Long- and Medium-term Scientific and Technological Development (2006–2020)”, “Biological Industry Development of The Eleventh Five-Year Plan”, “Policies to Speed up the Development of Biological Industry” and “The Twelfth Five-Year Plan”, which accelerated high-tech enterprises’ endeavours to become the mainstay in independent innovation. The policies thus initiated a focus on gene drugs and antibody R&D in 2006, while it was also the first time to affirm the long-term development of mAbs.

Up until 2009, four out of nine major projects had an involvement in the biological pharmaceutical field; at same time, the China Food and Drug Administration extended the duration of the state first-class new medicine to 12 years through the revision of two main regulations on new drug approval and technology transfer to encourage the development of innovative drugs, consequently streamlining R&D and manufacturing. The “Several Policies to Speed up the Development of the Biological Industry” program in 2009 expanded the financing channels and introduced risk investment to the fast-increasing investment of private industries. Details are shown in Table 3.

**Table 3:** Biopharmaceutical regulations and influences related to mAbs

Year	Title	Institute	Content	Aims	Influence/mAb
1987	Development Plan for Biological Products Career	Ministry of Health	Biopharmaceutical industry is given priority to develop the vaccine	To lay a foundation for further technology development	Focus on vaccine, mAb industry in China has not developed well
2006	National Program for Long- and Medium-term Scientific and Technological Development (2006–2020)	The State Council	Reaffirm a fact that biotechnology is an emerging technology and the focus of the future high technology industry tool of catching up is very important	To make enterprises to be innovators, cultivate a group of world-class scientists and endeavour to turn out a batch of influential breakthroughs	Middle and small enterprises cannot become the main body of innovations
2007	Biological Industry Development of the “Eleventh Five-Year Plan”	The Development and Reform Commission	The overall planning and deployment of biological industry. Four out of nine companies involved in biomedical field	To form a cluster and have local advantages	Strengthen the technical innovation ability construction, promote the achievements of transformation and the development of industrial agglomeration, advance cooperation with developed countries
2009	Several Policies to Speed-up the Development of the Biological Industry	The State Council	Biotech drugs should be developed for the treatment of common and serious diseases	To accelerate realization of the aim of fostering biopharmaceutical industry into a strategic pillar in industry	Promote the cooperation and restructure between business-to-business, enterprises and academies, expand the scale of the enterprises
2010	Decision about speeding up of cultivating and developing strategic emerging industries	The State Council	From the aspects of the fiscal and taxation financial policies to speed up the cultivation and development of strategic emerging industries	To take in a new round of economic and technology development commanding heights	Clearly fefine the position of antibody drugs and support the industry
2012	National Basic Medicine Catalogue	SFDA	Drugs in the list of essential medicines are to meet the needs of basic medical and health care The dosage form is appropriate, the price is reasonable, and can guarantee the supply, the public can have equitable access to medicine	To protect people’s health, to meet people's needs, and to make the country resources get the most reasonable use	mAbs gradually listed on the catalogue, expanded the market
2012	“Twelfth – five” Plan	The State Council	Discovery of new target, construction of humanized antibodies, development of therapeutic antibodies for major non-infectious diseases (malignancy, metabolic disease and autoimmune disease)	To carry out the innovation-driven development strategy, get output of major landmark achievements	Emerging industries were supported and advanced rapidly; enterprises are guided to speed up R&D of “Me-better” drugs
2013	The “Twelfth – Five Plan” of biological industry development	The State Council	Emphasize R&D of new drugs for major diseases, speed up the process of therapeutic antibody innovation, give support to develop antibody production industrialization	To get significant results in the field of antibody and reach world-class levels in a decade	Get special funds to support R&D, mAbs for anti-tumour advanced rapidly

Note: “SFDA” is the former name of the China Food and Drug Administration.

In summary, China implemented policies from three main perspectives: market, enterprises, and academies. Further, it made efforts to maintain a stable market environment and to establish a competitive system for mAbs. Additionally, the government provided aid to main business actors to encourage cooperation among themselves and networking with academies, which led to the beneficiaries eventually achieving positive results in a phase by phase basis. Being closely connected with the foundation of the mAb industry, policies also played an important role in pushing mAb development forward considerably. In the process of industrialization, R&D in mAbs had changed from the state of “following suit” to innovating and keeping updated not only in laboratory studies but also in manufacturing. Finally, in order to ensure that enterprises are the main body of innovation and that small- and medium-sized enterprises were involved in the development, effective long-term programs and actions specific to mAb development needs are expected to be taken by the State.

## Functions of mAb innovation system in China

**Figure 1:**
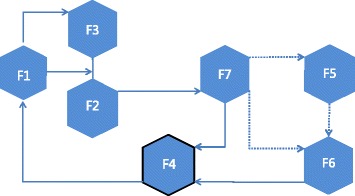
Overview of reinforcing cycles within an innovation system of mAb in China. F1, Entrepreneurial activities; F2, Knowledge development; F3, Knowledge diffusion through networks; F4, Guidance of the search; F5, Market formation; F6, Resource mobilisation; F7, Advocacy coalition.

Based on the results from our analysis, the function performance of the mAb innovation system in China can be summarized as follows (Figure 1):

- Entrepreneurial activity (F1) is prospering, with a value chain clearly dominated by foreign players since the last century. However, China’s mAb supply chain is growing rapidly and expected to become one of the top 10 largest in the near future.
- Knowledge development (F2) is being driven by incentives such as the R&D subsidies from the State and corporate R&D efforts, and innovation pressure such as fierce price competition from the leading countries and the need to improve antibody expression performance.
- Knowledge diffusion (F3) has been best served over the last 10 years through information exchange in networks and technology transfer between developed countries and China. Clearly, the mAbs leading enterprises like CPGJ were driving this growth and the national policies were channelling much of this demand towards domestic suppliers.
- Guidance of search (F4) has improved. The early transfer successes helped to highlight the need for independent innovation and competitive designs. As operators of mAbs, the enterprises had experienced a downside and had access to first-information of the strengths and weaknesses of their technologies.
- Market formation (F5) is not strong enough and there are many replacement drugs to share the market. However, high potential niche markets (export and globalization) have been explored.
- Resource mobilisation (F6) or resource supply situation has improved in that some resource categories are supported by the State (financial and industrial resources); however, for others there are still obstacles on the path to professional industrialization (human and infrastructural resources).
- Advocacy coalition or legitimacy (F7) has gained momentum due to the government policies of The Eleventh Five-year Plan in 2007 and the national projects named as the “973 Program” and the “863 Program”. The successes in building a domestic mAbs industry and the increase of the targets have reinforced the legitimacy of the technology furthermore.

In general, the virtuous circle in China starts with F4: ‘Guidance of the search’. The state fully realized the unique opportunities of mAb as an emerging technology and set the direction of mAb industry development in China. Motivated by the state encouragement, entrepreneurial activities (F1) were undertaken by different types of entrepreneurs. Then, targeted mAb technologies were developed (F2) and diffused (F3) through networking among firms and universities. In particular, in order to make further technology development possible and knowledge diffusion, the government established a state-level scientific research system and contributed to international cooperation. The rapid technology development and diffusion provided further advocacy coalition (F7) for mAb in China. In this case, the legitimacy supported by the State is beginning to stimulate market formation (F5) and mobilize several types of resources (F6), including finance, human resources, etc., into the mAb industry. Thus, a complete virtuous cycle of the mAb innovation system has been established and is operating in China.

To sum up the experience, the main force behind the development of mAb in China has been the strong political commitment to mAb technology, which activated other functions of the mAb innovation system. Positive feedback loops have been established with the legitimacy of this technology elevated with knowledge development and market formation. The regulatory mechanisms have successfully contributed to the emergence of a massive mAb innovation system in China.

## Discussion

To distinguish the characteristics of the mAb innovation system in China, a series of comparative discussions are presented herein. First, we compare the Chinese mAb innovation system with that of the United States, which is regarded as the leading mAb innovation system in the world. Second, we compare it with the mAb innovation system in India, which is rapidly catching up in the global frontier. Third, a comparison between mAb and pharmaceuticals in China is conducted. With these comparative analyses, a deeper understanding about how the mAb innovation system in China was founded will be obtained. After these comparative discussions, implications from this study will be presented to provide references for how to build innovation system to seize emerging technologies.

### Comparison of mAb development between the United States and China

For mAb industry development, the United States has significant advantages of conducting research and manufacturing products [44]. The mAb production capacity of the United States is ranked as the first in the world, drawing on the production bases rooted from its traditional pharmaceutical industry. There is large production capacity gap between China and the United States. In the technological aspect, mAb products in the United States belong to the “first-class technology” product class and are fully human. The United States has gathered the world’s top academic and research institutions. Viewed from the technology resource perspective, the United States has conducted its independent R&D. Patents can be transferred freely between different actors and converted with flexibility into commercialization [45], which is what China still lags behind.

Furthermore, actors in the United States and their activities are playing crucial roles in the progress of mAb industrialization; in some ways, even more than the State itself. With respect to the financial system, for instance, firstly, the combination of big pharma and venture capital is a basic pattern for the biotechnological entrepreneurship model in the United States. Secondly, there are several entrepreneur companies in the United States, who are growing fast as the leading power in mAbs. Thirdly, the mAb companies from the United States have enough capabilities to compete in the global market and to hold their leading market position. Finally, the federal government’s policy, plus special plans or projects of the various states, make policies flexible for mAb development and commercialization [46].

In summary, functions of the mAb innovation system in the United States work favourably to its knowledge development (F2) and research advancement guided by the ever-increasing high expectations (F4). The entrepreneurial activity (F1) is outstanding in leading to more knowledge formation (F2) and lobbying (F7) for better conditions. Clearly, the United States and China are moving in the same direction for technology development, but not enjoying the same status as competitors.

### Comparison of mAb development between India and China

In terms of corporate behaviour, the mAb actors in India cooperate more actively with other countries. For example, they have deep cooperation with the United States and Cuba to absorb mature mAb technologies. In terms of market positioning, India started by developing the overseas market and hit back to the domestic market with lower prices [47]. In terms of the financial policy, the government is not inclined to support enterprises since most of the national funding is allocated to the scientific research institutions. The main financial resources for enterprises come from venture capital. Scientific research institutions are still the main innovator of Indian mAb industry [46]. As for the technical route, China and India’s clinical trials both started using mouse antibodies, then transiting to human. With regards to the development strategy, they both went from imitation to innovation [48]. Concerning the industrial composition, both mAb industries are constituted mainly by middle- and small-sized enterprises and both countries are at the start-up stage. As for the development bottleneck, China and India are both restricted by technology and production capacity [45, 49].

However, the question remains as to why India’s development in the mAb industry is faster than that of China [50]. The key lies in the loose policy of generic drug and tight cooperation in the domestic and overseas endeavours. It also imitated the production–marketing model of pharmaceuticals. Since the revision of India’s patent law in 2005, India’ mAbs have occupied a larger market share around the world, laying the foundation for its internationalization [51]. India took advantage of the innovation ability of its leading companies to unite into one company [52], and built a Bio-Valley around Bombay bringing obvious advantages in market reaction [53].

In summary, the virtuous cycle of mAb innovation system in India starts with ‘entrepreneurial activities’ (F1). The actors tried to lobby the government to create legitimacy (F7) and make resource mobilisation (F6) but the practical results are disappointing. Then, they turned to improved behaviour (F1) to build up new overseas markets (F5). India and China are on the same technical route, but with different technologies and market strategies [54].

### Comparison between mAbs and pharmaceuticals in China

Pharmaceuticals in China have been developed and marketed since the 1950s. With regards to enterprise behaviour, the production of pharmaceuticals accounts for a large market share and represents a large industrial cluster. Further, there are various forms of cooperation on academic exchange and financial investments between these enterprises. With regards to market performance, while mAbs have to be promoted exclusively through academic channels, the pharmaceuticals industry in China has an existing mature market network and can be promoted through a variety of channels. The experience of the Chinese mAb industry compared with that of pharmaceuticals is summarized in Table 4.

**Table 4:** Comparison between mAbs and pharmaceuticals in China

	mAbs	Pharmaceuticals
Specificity	High	Very low
Side effect	Big (early products)	Very big
Scope of indications	Wide range	Wide range
Potential of drug transformation	Large	Large
Year	1980s	1950s
Objective	Prevention and control of major diseases	People’s basic life safety
Technology source	Transfer of key technology	All generic
Cross-disciplines	Biology: proteomics, genetic engineering	Combining with biology, traditional Chinese medicine
Level	Starting period	Mature period
Industry threshold	High investment threshold, high barriers of entry	Low investment threshold, low barriers of entry
Actor scale	Individual leading enterprise	Most of enterprises in China
Actor	Academies	Enterprises
Market strategy	Academic promotion	Hospital, pharmacy
Bottleneck	Large scale production	Hard to R&D, price controls strict, over capacity

At the technological level, domestic mAb products are diversified and developed as biosimilar to the foreign countries’ core technology, albeit with different targets. In contrast, pharmaceuticals can be manufactured completely as generics. As a consequence of lack of innovation investment in the domestic pharmaceuticals industry, a large number of redundant constructions appeared. Compared with other enterprises, the number of mAb enterprises is very small, and are all small- and medium-sized businesses. Their product lines and promotion strategies are undiversified, with less interaction with each other. Among pharmaceutical enterprises, there are some big and prestigious companies with product diversification and diversified sales channels [55]. The cooperation or merger between such enterprises occurs frequently. As a result, these two types of products have different performance profiles in the market. According to different technology levels, mAb have high technical barriers to entry and need high regulatory standards. On the other hand, for pharmaceuticals, their low regulatory standards and low entry barriers are likely to lead to the disorder of market competition. In addition, the funding mechanisms for pharmaceuticals and biopharmaceutical drugs such as mAbs are different in China [56]. In general, compared with pharmaceuticals, functions of mAbs start with entrepreneurial activities. Therefore, improving products and manufacturer capability (F2) is the approach to lobbying for better economic conditions in order to make further technology development possible (F7) and maintain fairer competition in the domestic market.

### Implications

This study on the mAb innovation system in China yields various management and policy implications. Compared with the development of mAbs in other countries, such as United States and India, their development in China is different. The virtuous circle of mAb innovation system in China starts with government’s strong guidance for industrial direction, and then moves to entrepreneurship and technology development and diffusion, both of which are strongly supported by the government. Such an innovation system experience has at least two important implications.

First, the experiences of mAb development in China imply that an innovation system could not be founded by a single actor. On the contrary, to establish an innovation system to seize the emerging technology, multiple types of actors need to be gathered and work collectively to ensure that an innovation system can be founded and managed in an efficient way. In the process, the government as a powerful actor, is always required to play the leading role at the earliest stage. However, to stimulate the internal dynamics of an innovation system, the government needs to transfer the leading role to entrepreneurial firms and research institutes in the next stages. This role-transferring process is very important for the maturity of an innovation system.

Second, the connection between innovation systems and research systems is crucial for the sustainable development of emerging technologies such as mAbs. As shown in this study, having realized the challenges and uncertainties of mAbs as a new emerging biopharmaceutical technology, China has made great investment in establishing a research system in its universities and research institutes. While technology transfer from other countries such as Cuba and the United States could provide opportunities to catch up with the international mAb frontiers in the short term, China is clearly conscious of the impact of its research system on its innovation system. With political guidance on encouraging and supporting linkages between firms and academic organizations, the connection between the research system and the innovation system has never been so accentuated.

## Conclusion

This study finds that an innovation system for mAb has been initially established in China to seize this emerging biotechnology opportunity. Such an innovation system was stimulated by the strong policy commitment from the government at the beginning but was later strengthened by entrepreneurial activities. MAb innovators in China are capitalizing on opportunities to participate in the development of an innovative drug value chain while strengthening their capabilities to interconnect and compete with established companies of developed countries. In the long run, the build-up of the research system for mAbs in China is expected to lead to more driving forces in the mAb innovation system.
